# Development of a Japanese Healthy Diet Index: The Fukushima Health Management Survey 2011

**DOI:** 10.3390/ijerph192214858

**Published:** 2022-11-11

**Authors:** Enbo Ma, Tetsuya Ohira, Seiji Yasumura, Mitsuaki Hosoya, Makoto Miyazaki, Kanako Okazaki, Masanori Nagao, Fumikazu Hayashi, Hironori Nakano, Eri Eguchi, Narumi Funakubo, Michio Shimabukuro, Hirooki Yabe, Masaharu Maeda, Hitoshi Ohto, Kenji Kamiya

**Affiliations:** 1 Health Promotion Centre, Fukushima Medical University, Fukushima 960-1295, Japan; 2 Department of Epidemiology, Fukushima Medical University School of Medicine, Fukushima 960-1295, Japan; 3 Radiation Medical Science Centre for Fukushima Health Management Survey, Fukushima Medical University, Fukushima 960-1295, Japan; 4 Department of Public Health, Fukushima Medical University School of Medicine, Fukushima 960-1295, Japan; 5 Department of Paediatrician, Fukushima Medical University School of Medicine, Fukushima 960-1295, Japan; 6 Department of Physical Therapy, Fukushima Medical University School of Medical Sciences, Fukushima 960-8516, Japan; 7 Department of Diabetes, Endocrinology, and Metabolism, Fukushima Medical University School of Medicine, Fukushima 960-1295, Japan; 8 Department of Neuropsychiatry, Fukushima Medical University School of Medicine, Fukushima 960–1295, Japan; 9 Department of Disaster Psychiatry, Fukushima Medical University School of Medicine, Fukushima 960-1295, Japan; 10 Research Institute for Radiation Biology and Medicine, Hiroshima University, Hiroshima 734-8553, Japan

**Keywords:** healthy diet index, dietary pattern, food frequency questionnaire, Fukushima health management survey

## Abstract

A novel healthy diet index for dietary quality can be used to assess food intake. After the Great East Japan Earthquake in 2011, the Fukushima Health Management Survey collected dietary data using a short-form food frequency questionnaire (FFQ). The current study included eligible participants (*n* = 64,909) aged 16–84 years who answered the FFQ in 2011. The year- and sex-specific dietary patterns were determined via principal component analysis. Based on the typical Japanese, juice/dairy, and meat patterns, healthy diet index (HDI) scores were assigned for food items, resulting in Spearman’s correlation coefficients of 0.730, −0.227, and −0.257, respectively. The mean (standard deviation) of the HDI scores (range: 1–18) were 9.89 (2.68) in men and 9.96 (2.58) in women. Older individuals, women, nonsmokers, those in good health and with regular physical exercise, and those who did not transfer residences had a high HDI score. In the confirmatory analysis, the adjusted odds ratio (95% confidence interval) of the highest vs. the lowest quartiles of HDI scores was 0.87 (0.80, 0.94) for overweight, 0.89 (0.81, 0.97) for large waist circumference, and 0.73 (0.66, 0.80) for dyslipidemia. The HDI score obtained using the FFQ can be applied to evaluate dietary profiles.

## 1. Introduction

High-quality diets are associated with a significantly low risk of noncommunicable diseases based on studies using the Healthy Eating Index (HEI), Alternate Healthy Eating Index, Dietary Approaches to Stop Hypertension (DASH), Mediterranean diet score, and Dietary Inflammatory Index (DII) [1,2,3,4,5,6]. The Japanese government has updated the Dietary Guidelines for Japanese and published the Japanese Food Guide Spinning Top in 2010 [7], in which the recommended food patterns are remarkably consistent with those of Canadian and American guidelines [8]. The overall diet quality indexes were not significantly better in predicting morbidity or mortality than individual dietary factors. However, they can help assess the extent to which individuals adhere to dietary guidelines [9]. No single food contains all essential nutrients. Therefore, different dietary sources must be used to ensure a balanced diet [10]. 

Dietary patterns were identified from previous studies on the Fukushima Health Management Survey (FHMS). Further, the vegetable (typical) pattern with traditional Japanese foods was found to be associated with a low risk of cardiovascular and kidney diseases [11,12]. In the prevention of noncommunicable diseases in clinical practice, dietary guidelines are helpful for health professionals. Moreover, dietary index scores are more accessible than dietary pattern scores directly derived from statistical models. 

A few studies have developed Japanese dietary quality scoring systems [13,14]. The current research aimed to develop a Japanese healthy diet index (HDI) using a short–form food frequency questionnaire (FFQ) in the FHMS, which can be used in other studies for assessing the dietary quality/food patterns or measuring correlations of dietary status/profile—as an independent causal factor or a confounder to other health risks—with interesting health outcomes. 

## 2. Materials and Methods

### 2.1. Study Participants

The FHMS was initially performed after the Great East Japan Earthquake in 2011. The Mental Health and Lifestyle Survey was conducted as part of the FHMS. In total, 73,431 (42.2%) participants responded to the survey questionnaires between July 2011 and March 2012 (2011 fiscal year, *n* = 210,189 target participants). The study protocol and baseline profiles are described in previous studies [15,16]. We included 70,139 individuals aged between 16 and 84 years who underwent the baseline mental health examination. This study was approved by the ethics committee of Fukushima Medical University, Japan (nos. 1316 and 1319; IPPAN 2020–239). 

### 2.2. Dietary Intake Assessment

The short-form FFQ with 19 food items was used to examine the food intake of participants within the previous 6 months. The FFQ was a modified version used in the Hiroshima and Nagasaki Life Span Study [17]. In its validation study, the food intake frequency measured using the FFQ was moderately correlated with food intake, which was measured using 24-h dietary recall data. The Spearmen correlation coefficient of fruit, milk, miso soup, beef/pork, rice, and bread intake was between 0.14 and 0.34 [17]. The 19 food items were divided into six food groups, which were as follows: non-juice fruits/vegetables (fruits, green, red and yellow, and light-colored vegetables), fruit/vegetable juices, meat (chicken, beef, pork, ham, and sausages), soybean products (fermented soybeans, soymilk, miso soup, tofu, and boiled beans), fish (raw and cooked), and dairy products (milk, yogurt, and lactobacillus drinks). The participants were asked about the frequently consumed individual food items (times/servings), with six response choices (none, <1 time/week, 1–2 times/week, 3–4 times/week, 5–6 times/week, and every day).

### 2.3. Statistical Analysis 

#### 2.3.1. Missing Dietary Data 

Following a previous study, we excluded participants with more than three missing responses to the FFQ (7.4% of 70,139 participants) [18]. Finally, 64,909 participants were included in the analysis; 11.6% and 4.0% had one and two missing answers to the FFQ, respectively. We imputed these missing values as per sex using the median value of the food intake frequency from available answers [18]. Using this method, the validity of further results was not affected.

#### 2.3.2. Polychoric Coefficients for Food Items

As the FFQ was a semi-questionnaire without information on the size portions of food intake, we could not compute the amount of food or nutrients. To assess food intake times/servings, we calculated the intake by using the daily midpoint of the frequency category. Consequently, food intake was recorded as 0, 0.017, 0.214, 0.5, 0.786, and 1 serving per day [18]. Because the food items were ordinal numerical variables with a certain distance between each category, we calculated the polychoric correlation coefficients for each pair of food frequencies. All paired polychoric correlation coefficients were significant (*p* < 0.0001), except those between fruit and ham (*r* = −0.0074, *p* = 0.083), miso soup and fruit juice (*r* = 0.0059, *p* = 0.214), and miso soup and vegetable juice (*r* = 0.0004, *p* = 0.926). 

#### 2.3.3. Derivation of Dietary Patterns

**Figure 1:**
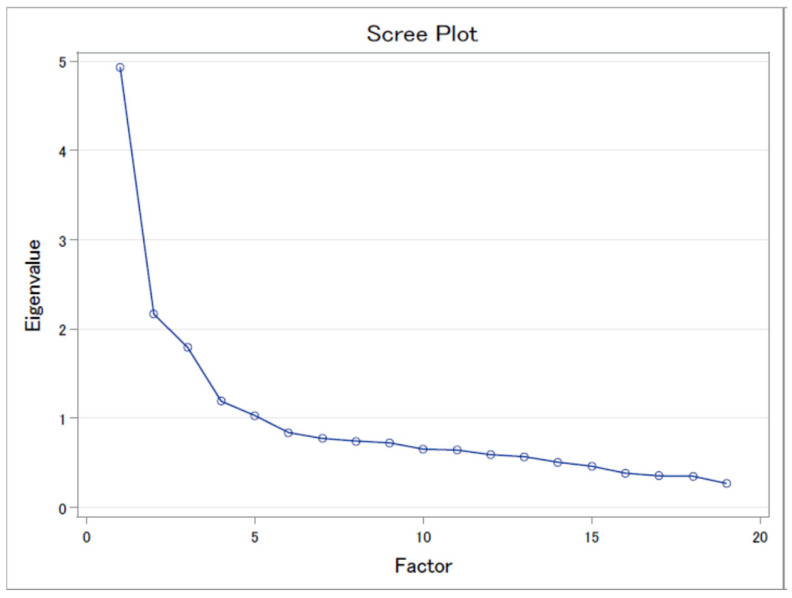
Scree plots of eigenvalues in the principal component analysis of food frequencies, Fukushima Health Management Survey, 2011.

Based on the polychoric correlation coefficients of the food items, the dietary patterns were derived using the principal component analysis. The scree plot was shown in Figure 1. The varimax rotation of the identified dietary patterns was applied to improve their interpretability. We selected three factors mainly according to eigenvalues (>1.5), scree plots, and factor interpretability, and considered food items with absolute factor loadings of ≥0.3 to account for each component [19]. The cumulative variance explained by three factors was 46.8%. We labeled the derived dietary patterns as typical Japanese, juice/dairy, and meat based on food items with high factor loadings in each dietary pattern (Table 1). To reflect how closely a diet resembles each identified pattern, the pattern-specific scores were calculated as the sum of the products of the factor loading coefficients and the standardized intake of food items [20]. 

#### 2.3.4. HDI

The Osaki cohort study developed the Japanese Diet Index, which assigned scores by identifying eight typical Japanese foods [21]. Using the same method to calculate the dietary index [21,22], we assigned one score for the food intake higher than the median in the typical Japanese pattern and one score for the food intake lower than the median level in the juice/dairy or meat pattern. The intake of boiled beans and fruits had factor loadings of >0.3 in the typical Japanese and juice/dairy pattern. Hence, when calculating the HDI for boiled beans and fruits, a score of 1 was assigned if the intake frequency was more than the median, while 0.5 was assigned if the intake frequency was less than the median. 

The Spearmen correlation coefficients were used to examine participants between the sum of dietary index scores and pattern-specific scores. The Cronbach’s coefficient alpha between the HDI and pattern-specific scores was assessed, with values higher than 0.70, which indicated good reliability (internal consistency) [23]. 

To evaluate the dietary status by the HDI in the study population, the least-square means and their difference in sociodemographic characteristics were computed using multivariable regression models. With linking to the individuals’ health checkup information in 2011 (*n* = 34,382), the confirmatory analysis for the associations of the HDI scores with cardiometabolic risk was conducted. The odds ratio (OR) and 95% confidence interval (CI) were calculated with the lowest quartile of the HDI scores as the reference.

All data were analyzed using the SAS statistical software package version 9.4 for Windows (SAS Institute, Cary, NC, the USA). Two-sided *p* values were reported, and the significance level was set at <0.05.

## 3. Results

Table 1 shows the food groups and factor loadings of the three dietary patterns. The typical Japanese diet pattern included vegetables (white, green, red, and yellow), fish, fruits, bean products (tofu, fermented and boiled beans, and miso soup), and rice. The juice/dairy pattern included vegetable juice, fruit juice, yogurt, soymilk, fruit, milk, boiled beans, and bread. The meat pattern included chicken, beef/pork, ham/sausage, and bread.

Table 2 depicts the healthy diet index scores assigned for each food item/group toward the typical Japanese, juice/dairy, and meat patterns using the median intake frequency as the cutoff value. 

**Figure 2:**
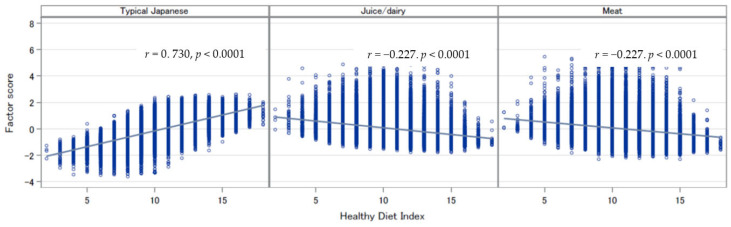
Correlations between the Health Diet Index scores and the dietary pattern scores.

Figure 2 shows that the HDI scores were positively correlated with the typical Japanese pattern scores and negatively associated with the juice/dairy and meat pattern scores for all participants. The standardized Cronbach’s coefficient alpha between the HDI and pattern-specific scores in all participants were 0.836, −0.482, and −0.564, respectively. The Spearman correlation coefficients between the index scores and the three pattern scores were 0.747, −0.260, and −0.257 in men and 0.720, −0.213, and −0.260 in women (*p* values < 0.0001), respectively.

The HDI scores ranged 1–18. The mean HDI (standard deviation) was 9.93 (2.62) in all the participants, 9.89 (2.68) in men, and 9.96 (2.58) in women. Women had a higher HDI than men (*p* < 0.0001). Table 3 shows the HDI scores (least-square means and standard error) obtained according to the population’s social and demographic characteristics. The HDI scores significantly differed in terms of sociodemographic factors. Older individuals, women, nonsmokers, alcohol drinkers, those who had low education levels, those in good health and had regular physical exercises, and who did not change residence after the disaster had a high HDI score. After stratifying according to sex, a similar significance was observed, except for the HDI score of alcohol drinkers in women.

Table 4 shows that higher the HDI score, lower the risk of being overweight and having a large waist circumference. The highest diet index category had a 27% lower risk of dyslipidemia. Additionally, the age–sex and multivariable-adjusted OR (95% CI) in the highest vs. the lowest quintile of the HDI scores was 0.69 (0.62, 0.75) and 0.70 (0.63, 0.78) for low-density lipoprotein cholesterol (≥140 mg/dL), and 0.78 (0.69, 0.89) and 0.83 (0.73, 0.94) for triglycerides (≥150 mg/dL), respectively.

## 4. Discussion

According to the identified factor loadings, we identified three dietary patterns in this population: the typical Japanese, juice/dairy, and meat. Subsequently, we assigned HDI scores using the median frequency of food intake in the typical Japanese diet pattern. The HDI scores were moderate to strongly correlated with the dietary pattern scores; and conveniently elucidated the diversity of the HDI in terms of sociodemographic factors, such as age, health condition, physical activity, and transfer of residence in the study population. Further, we observed significant inverse associations between the HDI scores and cardiometabolic risk, e.g., overweight and dyslipidemia, by linking to a health checkup dataset.

Dietary quality scores were developed mainly based on dietary guidelines, Mediterranean diet, foods, or nutrients [9]. Several healthy food indexes, such as the HEI [24], DASH [4], nutrient-rich food index [25], and DII [26], had common elements based on nutrient content assessed using the scoring algorithm. However, the Mediterranean diet score, which was established by Trichopoulou et al. [22], was the simplest. Further, its method was applied in this study. 

In Japanese populations, diet quality scores have been developed or adapted accordingly. The Osaki Study has developed a Japanese diet index score (range: 0–9) using the FFQ with 39 food items, which included rice, miso soup, seaweed, pickles, green and yellow vegetables, fish, green tea, beef and pork, and coffee [14,21]. The variance explained by the three dietary patterns in our study (46.8%) was higher than that in the Osaki Study (26.1%) [21]. Previous studies on the Japanese diet index have reported the positive effect of a healthy diet on functional disability in elderly individuals [21], those with a longer survival time [27], and those with a decreased risk of all-cause and cardiovascular disease (CVD)-related mortality [28]. The Japan Collaborative Cohort Study revealed that higher pro-inflammatory scores were associated with elevated serum C-reactive protein levels [29]. Previous studies that used dietary quality scores based on the Japanese dietary guidelines showed that young women had a low risk of total mortality and CVD-related mortality [13], small waist circumference, and decreased concentrations of low-density lipoprotein cholesterol [30]. A Japanese three-generation study evaluated significant protective associations between depression and diet quality score [31]. 

Compared with dietary pattern scores, a dietary food index is more convenient to use when evaluating individual dietary quality. Dietary patterns closely match eating behaviors and resemble the synergistic effects of multiple food groups in a specific population [32]. However, they may not reflect an optimal diet and are barely replicable in other populations [2]. Unlike a dietary pattern, which has difficulty in describing the protective and negative effects, as individual pattern scores calculated both have negative and positive values, the HDI incorporation of different patterns has only positive values, but covers both directions of the patterns’ effect, i.e., the higher the HDI, the higher diet quality or, the healthier dietary habits. Based on dietary patterns, rather than specific nutrients, the dietary index reflects adherence to an ideal diet. Thus, it can be helpful for health professionals when communicating with different individuals [2]. In this study we assigned scores using the median of food intake frequency while considering the food items across different patterns. Notably, an imbalance of HDI scores to the direction of each diet pattern, with higher scores weighted in one pattern, results in lower correlation coefficients. With novel scientific evidence, the HDI can be developed by adding more food items [24]. 

FFQs have different food items, and they are used to evaluate different populations. Therefore, the identified dietary patterns may differ. For example, the dietary patterns in Japanese populations comprise a combination of dietary staples, side dishes, and soups [33]. The patterns derived in our research had dietary categories similar to those in other studies [34]. The typical Japanese pattern is in accordance with the traditional Japanese [21,35] or healthy pattern [36,37] and the non-typical Japanese diet patterns with the high dairy and animal food [21,38], bread-dairy and animal food [35], or bread and western pattern [36]. In addition, some studies have explored dietary patterns by adding alcoholic drinks, tea, or coffee [21,39], or identified more patterns, such as the dessert pattern [36]. Although our study used the short-form FFQ, the main food groups were included in this study population. Therefore, the HDI scores based on three-factor dietary patterns may be sufficient to cover the prominent food groups and reflect diet quality.

Similar to other studies that considered sociodemographic factors, smokers had negative typical Japanese pattern scores, and their scores were lower than those of nonsmokers [38,40]. Importantly, women are more likely to follow the typical Japanese pattern [41]. Residents with poor health conditions had lower HDI scores than those with good health conditions. Participants with a higher educational level and non-alcohol drinkers tended to have lower HDI scores. This may be attributed to the interaction effects of occupation and other dietary habits or food intake, which need further validation. Notably, better dietary habits, food availability and supply, and continuous nutritional support are important for people with difficult living conditions, particularly those who are vulnerable [38,42].

After the Great East Japan Earthquake on 11 March 2011, residents in radiation exposure areas were advised to move to the government-designated evacuation zone. Considering that several evacuees could have changed their lifestyle (particularly diet and physical activity), the risk of developing lifestyle diseases (e.g., CVD and metabolic syndrome) could have increased [43,44]. A study conducted in Miyagi prefecture, another area severely damaged by tsunami, identified a prudent pattern and meat pattern, and the prudent pattern was similar to the typical Japanese pattern in our study [38]. Participants who did not change residence (including non-evacuees) had higher HDI scores than their counterparts. The FMHS reported that living in nonhome conditions is associated with a poor dietary intake of fruits and vegetables, meat, and soybean and dairy products [18]. This finding might be attributed to limited room space or kitchen equipment, which causes difficulties in preparing daily balanced meals [18,38,45]. After the disaster, it was essential to monitor the long-term diet quality of Fukushima residents.

The strength of the current study was that it included a large population of varying ages in the disaster area. However, it also had some limitations. First, approximately 27% of evacuees responded to the FHMS [43]. The FHMS was designed to monitor the health status of residents in the radiation-exposed areas. Thus, the representativeness of this study on dietary patterns might not be generalizable to all individuals in the whole prefecture or the country’s general population. Second, this study transformed the HDI from dietary patterns derived from the adapted FFQ alone (19 food items without nutrient and energy-adjusted evaluation). As the FFQ also had ordinal frequency variables, it may be difficult to convert them to small numbers of variables with minimal loss of original information [46]. The internal consistency of the FFQ data in the study population was high for the typical Japanese pattern but not for the juice/dairy and meat patterns. Although polychoric coefficients were computed for discrete food variables, the HDI derived from dietary patterns with relatively lower explained variance might not be applicable in classifying broad food intake. Therefore, a more comprehensive HDI with a higher number of food items could not be developed. For example, energy density might be a simple alternative to dietary quality [47]. Wheat-based food, a favorite dish among Japanese, was not included in the FFQ. Moreover, based on previous dietary guidelines, only a limited amount of salt is recommended [7]. However, in this research, salt intake could not be assessed. Third, the participants reported their food consumption. Hence, their actual dietary intake might be underreported, which could result in nonrandom misclassifications [19]. Furthermore, the associations between HDI scores and cardiometabolic risk factors/events were carried out in a cross-sectional manner. The significant associations may not be causal relations; thus, the performance of the HDI needs to be examined in populations with follow-up outcomes. Nevertheless, further studies on replicability and validity should be conducted and compared to other available healthy indexes.

## 5. Conclusions

We developed the HDI based on the dietary patterns derived from the short-form FFQ in the FHMS, which incorporated different components of the typical Japanese and non-Japanese dietary patterns. This tool could be applied for the assessment and education of healthy food intake in the community or among different individuals. The index may also be used for the surveillance of individual nutritional food as a tool to assess gaps with respect to food guidelines. Further, the HDI score was found to be associated with social and demographic factors. Nevertheless, further studies on the use of HDI in evaluating the impact of overall lifestyle on health outcomes must be performed. 

## Figures and Tables

**Table 1:** Factor loadings of dietary patterns identified via principal component analysis, Fukushima Health Management Survey, 2011 (*n* = 64,909).

	Three Patterns		
Foods and Food Groups	Typical Japanese	Juice/Dairy	Meat
White vegetable	**0.74**	0.10	0.19
Miso soup	**0.72**	−0.09	−0.08
Green vegetable	**0.70**	0.18	0.18
Tofu	**0.70**	0.16	0.03
Red/yellow vegetable	**0.68**	0.25	0.25
Rice	**0.60**	−0.22	0.03
Fish	**0.60**	0.05	0.16
Fruit	**0.55**	**0.41**	−0.07
Fermented bean	**0.50**	0.17	−0.14
Vegetable juice	−0.02	**0.75**	0.08
Fruit juice	−0.04	**0.72**	0.15
Soymilk	0.08	**0.62**	0.01
Yogurt	0.27	**0.57**	−0.02
Boiled bean	**0.48**	**0.45**	0.01
Milk	0.22	**0.42**	0.04
Bread	−0.15	**0.32**	**0.32**
Beef/pork	0.15	−0.04	**0.79**
Chicken	0.14	0.09	**0.74**
Ham/sausage	0.04	0.07	**0.73**
Variance explained (%)	4.20	2.67	2.03

Loadings of an absolute value of > 0.30 are shown in bold.

**Table 2:** Assignments of healthy diet index scores according to the median intake of food items.

Food Groups		Score Assigned		Score Assigned
White vegetable	<median	0	≥median	1
Green vegetable	<median	0	≥median	1
Tofu	<median	0	≥median	1
Miso soup	<median	0	≥median	1
Red/yellow vegetable	<median	0	≥median	1
Fish	<median	0	≥median	1
Fermented bean	<median	0	≥median	1
Fruit	<median	0.5	≥median	1
Boiled bean	<median	0.5	≥median	1
Rice	<median	0	≥median	1
Vegetable juice	<median	1	≥median	0
Fruit juice	<median	1	≥median	0
Yogurt	<median	1	≥median	0
Soybean milk	<median	1	≥median	0
Bread	<median	1	≥median	0
Milk	<median	1	≥median	0
Beef/pork	<median	1	≥median	0
Ham	<median	1	≥median	0
Chicken	<median	1	≥median	0

**Table 3:** Healthy diet index scores obtained according to the sociodemographic characteristics, Fukushima Health Management Survey, 2011.

	All Participants (*n* = 57,824)			Men (*n* = 26,028)			Women (*n* = 31,796)		
	%	Mean *	SE	%	Mean *	SE	%	Mean *	SE
Sex									
Men		9.56	0.03	–			–		
Women		9.74	0.03	–			–		
*p*-value **	<0.0001								
Age (years)									
16–29	12.2	8.09	0.04	11.5	8.03	0.06	12.8	8.13	0.05
30–39	14.6	8.74	0.04	13.5	8.63	0.05	15.6	8.80	0.05
40–49	13.5	9.15	0.04	12.7	9.03	0.05	14.2	9.20	0.05
50–59	18.6	10.04	0.03	18.7	9.82	0.05	18.5	10.15	0.05
60–69	22.2	10.77	0.03	24.4	10.59	0.04	20.4	10.84	0.05
70–84	18.9	11.14	0.03	19.3	11.04	0.05	18.6	11.14	0.05
*p*-value **	<0.0001			<0.0001			<0.0001		
Educational level									
Elementary/junior high school	21.4	9.70	0.03	23.2	9.68	0.04	19.9	9.64	0.05
High school	51.7	9.61	0.03	52.5	9.50	0.04	51.0	9.63	0.04
Vocational college	17.7	9.62	0.03	11.1	9.49	0.05	23.2	9.74	0.05
Undergraduate/graduate	9.2	9.68	0.04	13.3	9.41	0.05	5.9	9.82	0.07
*p*-value **	0.002			<0.0001			0.001		
Health condition									
Good	18.6	9.83	0.03	22.0	9.72	0.05	15.9	9.88	0.05
Normal	63.1	9.65	0.03	60.6	9.53	0.04	65.2	9.69	0.04
Poor	18.3	9.48	0.03	17.4	9.32	0.04	19.0	9.56	0.05
*p*-value **	<0.0001			<0.0001			<0.0001		
Smoking									
No	55.8	9.71	0.03	27.4	9.51	0.04	79.1	9.83	0.04
Former	21.8	9.69	0.03	37.1	9.60	0.04	9.3	9.67	0.05
Current	22.4	9.57	0.03	35.5	9.46	0.04	11.6	9.63	0.05
*p*-value **	<0.0001			0.001			<0.0001		
Alcohol drinking									
No	50.9	9.61	0.02	29.6	9.39	0.04	68.3	9.73	0.03
Former	3.3	9.57	0.06	5.0	9.45	0.07	2.0	9.62	0.10
Current	45.8	9.78	0.02	65.5	9.72	0.03	29.7	9.77	0.03
*p*-value **	<0.0001			<0.0001			0.169		
Physical activity									
Everyday	13.6	9.89	0.04	16.4	9.78	0.05	11.4	9.92	0.05
2–4 times/day	19.1	9.69	0.03	19.1	9.55	0.05	19.0	9.74	0.05
1 time/day	13.8	9.53	0.03	14.7	9.37	0.05	13.0	9.63	0.05
No	53.5	9.51	0.03	49.8	9.39	0.04	56.6	9.55	0.04
*p*-value **	<0.0001			<0.0001			<0.0001		
Depression									
Weak (K6 < 13)	85.8	9.68	0.02	88.2	9.53	0.03	83.8	9.74	0.04
Strong (K6 ≥ 13)	14.2	9.63	0.03	11.8	9.51	0.05	16.2	9.68	0.05
*p*-value **	0.086			0.609			0.07		
Change of residence									
Own/relatives’ house	54.1	9.76	0.03	53.2	9.66	0.04	54.9	9.78	0.04
Shelter/temporary/rent	45.9	9.55	0.03	46.8	9.39	0.04	45.1	9.64	0.04
*p*-value **	<0.0001			<0.0001			<0.0001		

SE, standard error; * least-square means of scores; ** Difference of scores in each category based on multivariable linear regression (without missing values).

**Table 4:** Odds ratios and 95% confidence intervals between the health diet index scores and cardiovascular risk, Fukushima Health Management Survey, 2011 (*n* = 34,832).

	Health Diet Index Score (Quartiles)				*p* Trend
	Q1 (Low)	Q2	Q3	Q4 (High)	
Index score	<8	8–<10	10–<12	≥12	
Number of participants	6567	8403	10,482	9380	
Overweight (BMI ≥ 25 (kg/m^2^)	2023	2636	3411	3066	
Model 1	1.00 (Reference)	0.92 (0.86, 0.99)	0.89 (0.83, 0.96)	0.81 (0.75, 0.87)	<0.001
Model 2	1.00 (Reference)	0.95 (0.88, 1.02)	0.93 (0.86, 1.00)	0.87 (0.80, 0.94)	<0.001
Large waist circumference (men ≥ 85 cm/women ≥ 90 cm)	1587	1028	2704	3291	
Model 1	1.00 (Reference)	1.04 (0.96, 1.02)	0.98 (0.91, 1.06)	0.99 (0.92, 1.07)	0.411
Model 2	1.00 (Reference)	0.99 (0.91, 1.09)	0.91 (0.83, 0.99)	0.89 (0.81, 0.97)	0.001
Hypertension *	1674	2887	4600	4984	
Model 1	1.00 (Reference)	0.94 (0.80, 1.11)	0.91 (0.80, 1.04)	1.01 (0.89, 1.15)	0.98
Model 2	1.00 (Reference)	0.92 (0.78, 1.08)	0.89 (0.78, 1.02)	0.99 (0.87, 1.13)	0.936
Diabetes mellitus **	406	699	1013	1138	
Model 1	1.00 (Reference)	0.73 (0.57, 0.95)	0.73 (0.57, 0.93)	0.79 (0.62, 1.00)	0.221
Model 2	1.00 (Reference)	0.77 (0.59, 1.00)	0.77 (0.60, 0.99)	0.85 (0.67, 1.10)	0.571
Dyslipidemia #	2856	3979	5546	5252	
Model 1	1.00 (Reference)	0.84 (0.77, 0.92)	0.81 (0.74, 0.88)	0.70 (0.64, 0.77)	<0.0001
Model 2	1.00 (Reference)	0.85 (0.77, 0.92)	0.82 (0.75, 0.89)	0.73 (0.66, 0.80)	<0.0001

BMI, body mass index; * hypertension, diastolic blood pressure ≥ 90 mmHg, systolic blood pressure ≥ 140 mmHg, or using antihypertensive medications; ** diabetes mellitus, fasting blood glucose ≥ 126 mg/dL, hemoglobin A1c ≥ 6.5%, or use of insulin; # dyslipidemia, high-density lipoprotein cholesterol < 40 mg/dL, low-density lipoprotein cholesterol ≥ 140 mg/dL, triglycerides ≥150 mg/dL, or use of antidyslipidemia therapy. Model 1, adjusted for age (16–29, 30–39, 40–49, 50–59, 60–69, 70–84) and sex. Model 2, further adjusted for smoking history (no, former, current, missing), alcohol drinking (no, former, current, missing), physical activity (none, once/day, 2–4 times/day, every day, missing), education (elementary and junior high school, high school, vocational college, university and above, missing), depression (K6 < 13, K6 ≥ 13, missing), health status (poor, normal, good, missing), and history of stroke (no, yes, missing), heart disease (no, yes, missing), cancer (no, yes, missing), hypertension (no, yes, missing), diabetes mellitus (no, yes, missing), or dyslipidemia (no, yes, missing).

## Data Availability

The data presented in this study are available from the corresponding author upon request. However, the data are not publicly available to prevent breaking confidentiality.
